# An Unusual Simultaneous Existence of Parathyroid Carcinoma and Papillary Thyroid Carcinoma: Case Report and Review of Literature

**DOI:** 10.1155/2020/2128093

**Published:** 2020-03-31

**Authors:** César Ernesto Lam-Chung, Diana Lizbeth Rodríguez-Orihuela, Jazmín De Anda González, Armando Gamboa-Domínguez

**Affiliations:** ^1^Department of Endocrinology and Metabolism, Instituto Nacional de Ciencias Médicas y Nutrición Salvador Zubirán, Vasco de Quiroga 15, Sección XVI, Tlalpan, México City 14000, Mexico; ^2^Department of Pathology, Instituto Nacional de Ciencias Médicas y Nutrición Salvador Zubirán, Vasco de Quiroga 15, Sección XVI, Tlalpan, México City 14000, Mexico

## Abstract

Synchronous parathyroid and papillary thyroid carcinoma are extremely rare. To our knowledge, only 15 cases have been reported in the last four decades. We describe a 50-year-old female without significant past medical or family history and no previous trauma presented with left heel pain that prompted her to seek medical attention. Physical examination was notable for a painless nodule at the left thyroid lobe. Laboratory evaluation showed a serum calcium level of 14.3 mg/dL (8.6–10.3 mg/dL) and intact parathyroid hormone level of 1160 pg/mL (12–88 pg/mL). 99Tc-sestamibi dual-phase with single-photon emission computed tomography fused images showed increased uptake at the left-sided inferior parathyroid gland. Neck ultrasound showed a 1.4 cm heterogeneous nodule in the middle-third of the left thyroid gland and a solitary 1.9 cm vascularized and hypoechoic oval nodule that was considered likely to represent a parathyroid adenoma. Due to its clinical context (severe hypercalcemia and very high levels of PTH), parathyroid carcinoma (PC) was suspected although imaging studies were not characteristic. The patient underwent en bloc resection of the parathyroid mass and left thyroid lobe and central neck compartment dissection. Pathology analysis revealed classical papillary thyroid carcinoma of classical subtype and parathyroid carcinoma. Immunohistochemical staining was positive for cyclidin D1 and negative for parafibromin. High clinical suspicion is required for parathyroid carcinoma diagnosis in the presence of very high level of parathyroid hormone, marked hypercalcemia, and the existence of any thyroid nodule should be approached and the coexistence of other carcinomas should be considered.

## 1. Introduction

Less than 1% of all cancers and primary hyperparathyroidism (PHPT) can be attributed to parathyroid carcinoma (PC) [1, 2]. The vast majority of cases are sporadic, whereas the rest occur in the context of genetic syndromes such as multiple endocrine neoplasia type 1 (MEN1) or hyperparathyroidism-jaw tumor syndrome (HPT-JT) [3, 4]. It has equal frequency in both genders and usually occurs during the fifth decade of life [1, 2]. Very high serum levels of intact parathyroid hormone (iPTH) and severe hypercalcemia are the main biochemical findings and clinical manifestations are due to its excessive secretion [5, 6]. It is usually diagnosed postoperatively with histology assessment given its absence of specific clinical and biochemical features [2].

Papillary thyroid cancers (PTCs) account the majority (80–85%) of thyroid cancer cases, specifically papillary thyroid microcarcinomas (PTMs) [7–9]. Radiation exposure and genetics are the known risk factors for PTCs. However, only 5% of PTC cases account for familial PTC [10]. It occurs predominantly in female, and the median age at presentation is 50 years [11]. An asymptomatic thyroid mass is usually the main clinical finding, and it can be accompanied with or without cervical lymph node enlargement. However, vocal cord paralysis and tracheal compression can be present in about 20% of PTC cases, and it is manifested as hoarseness and dysphagia [12]. Biochemical tests are of limited usefulness because most patients have normal thyroid function [13].

The coexistence of thyroid and parathyroid carcinoma is extremely infrequent. Here, we describe a patient with this unusual presentation.

## 2. Case Presentation

A 50-year-old female without significant past medical or family history and no previous trauma presented with pain on her left heel that prompted her to seek medical attention. Initial evaluation was notable for serum concentration of calcium 14.3 mg/dL (normal range 8.6–10.3 mg/dL), alkaline phosphatase of 339.4 U/L (normal range 34–104 U/L), creatinine of 1.8 mg/dL (normal range 0.3–0.7 mg/dL), and iPTH level of 1160 pg/mL (normal range 12–88 pg/mL). Biochemical data of the patient are summarized in Table 1. Because of the concern for chronic kidney disease, a renal ultrasound was performed and showed both kidney medullary nephrocalcinosis and thinning of the renal parenchyma. Bone densitometry revealed osteoporosis at the anteroposterior spine (T score −2.7) and femoral neck (T score −2.9) and osteopenia at the total hip (T score −2.3). A presumptive diagnosis of parathyroid carcinoma (PC) was made and referred to endocrinology department for further evaluation. Physical examination revealed a hardened and painless nodule at the left thyroid lobe without vocal cord paralysis. Thyroid function test values were within the reference ranges.

**Figure 1:**
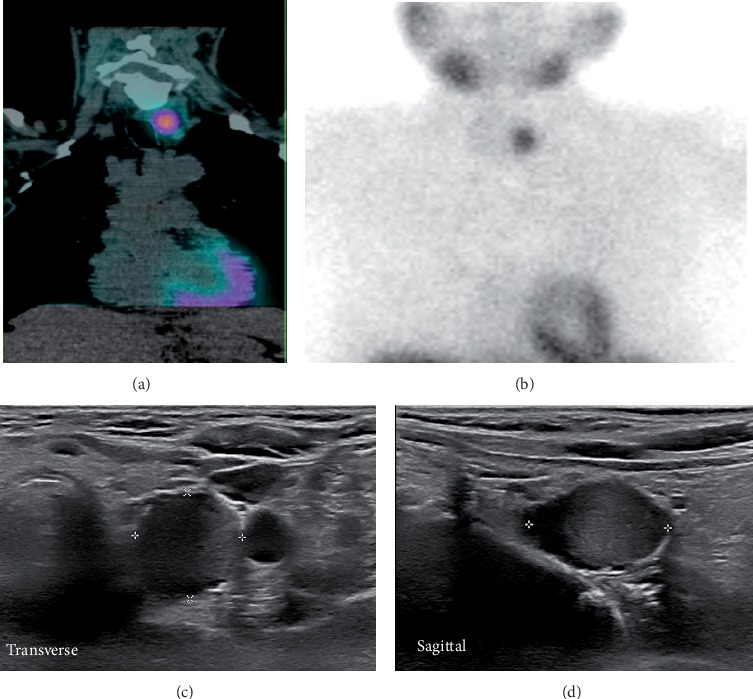
(a, b) 99mTc-sestamibi dual-phase with single-photon emission computed tomography/computed tomography fused images, showing uptake within middle to inferior left lobe of the thyroid. (b, c) Ultrasonograms revealing an oval-shaped hypoechoic solid nodule (1.40 × 1.39 × 1.92 cm) at the same area.

**Figure 2:**
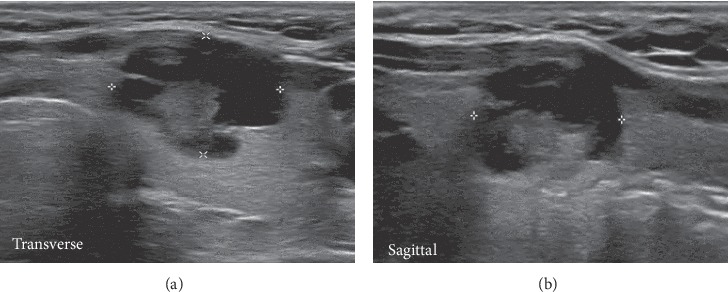
(a, b) Ultrasonograms (transverse and sagittal views), demonstrating a fairly oval-shaped solid isoechoic nodule with mixed solid and cystic components (1.38 × 1.14 × 1.36 cm) in the middle portion of the left thyroid lobe.

A 99mTc-sestamibi dual-phase fusion imaging with single-photon emission computed tomography/computed tomography (SPECT/CT) was performed, and a possible left-sided inferior parathyroid was identified (Figure 1(a)). Neck ultrasonography showed a 1.4 cm heterogeneous nodule and central vascularity in the middle-third of the left thyroid gland (Figures 2(a) and 2(b)). At the same area depicted by SPECT/CT, a 1.9 cm vascularized and hypoechoic oval nodule was present and considered likely to represent a parathyroid adenoma (Figure 1(b)). Due to its clinical context (severe hypercalcemia and very high levels of PTH), PC was suspected although imaging studies were not characteristic. At surgical intervention, a normal-appearing inferior parathyroid gland was identified and preserved in situ. An en bloc resection of the parathyroid mass and left thyroid lobe and central lymph node compartment dissection was performed. On the fourth postoperative day, the iPTH value was 84.6 pg/mL.

**Figure 3:**
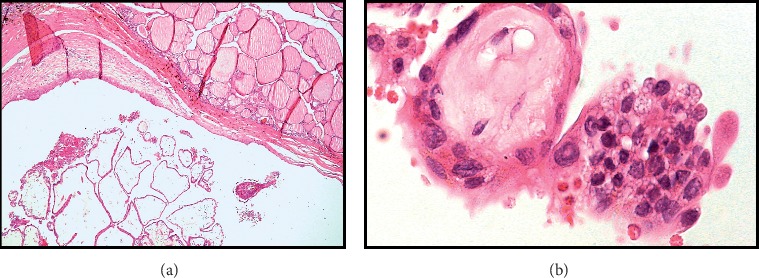
(a) Histologic appearance of the 1.3 cm papillary carcinoma in the left thyroid lobe, demonstrating a cystic neoplasia with a papillary pattern in transition with residual thyroid normal tissue (hematoxylin-eosin stain original magnification ×4). (b) Histopathological section showing typical features of papillary carcinoma: fibrovascular stalk covered with follicular cells depicting eosinophilic cytoplasm follicular cells and oval nuclei (^*∗*^) with pseudoinclusions (blue arrow) (hematoxylin-eosin stain original magnification ×40).

**Figure 4:**
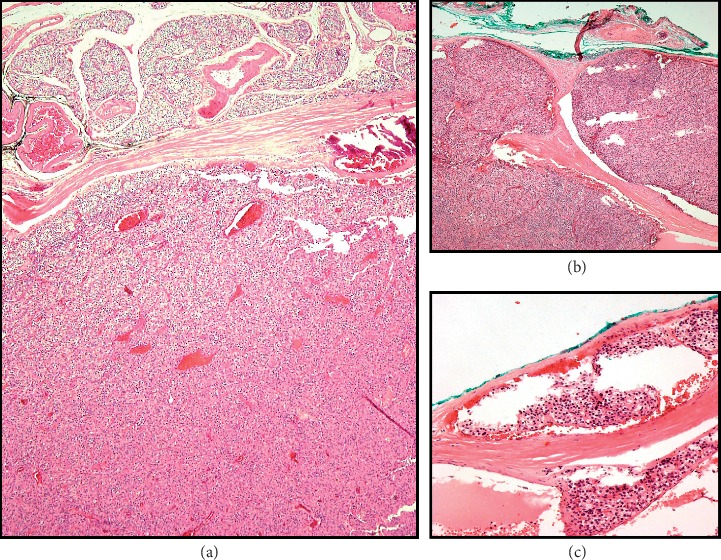
(a) Histology appearance of parathyroid carcinoma in transition with papillary thyroid carcinoma removed from the left thyroid lobe. The neoplastic cells are represented by the monomorphic cellular proliferation with discrete atypia and mitosis (hematoxylin-eosin stain original magnification ×4). (b) Histopathologic section demonstrating capsular invasion and (c) vascular invasion (hematoxylin-eosin stain original magnification ×10).

Gross examination of the surgical specimen revealed a 4.8 × 2.6 × 2.3 cm left thyroid lobe and a 2.4 × 1.8 × 1.4 cm left superior parathyroid gland. Histological examination revealed a 1.3 × 1.2 cm mass at the left thyroid lobe consistent of PTC of classical subtype (Figure 3) and PC, 2.5 cm in widest tumor dimension, with capsular and vascular space invasion (Figure 4). Immunohistochemical staining was positive for cyclidin D1 and negative for parafibromin. Ki-67 index was 2%. Six examined lymph nodes from central compartment were negative for metastasis. Her thyroid carcinoma was staged pT1bN0M0 (TNM AJCC 2018).

On the sixth postoperative day, the patient presented hypocalcemic symptoms (calcium 6.9 mg/dL) consistent with hungry bone syndrome. She received intravenous and oral calcium and vitamin D supplementation. Subsequently, she was discharged with oral calcium treatment and remained well, with the latest serum calcium level being 8.6 mg/dL (6 weeks postoperatively).

## 3. Discussion

The simultaneous presence of thyroid and parathyroid carcinoma is extremely rare with only 15 documented cases [14–30] in the last 41 years, and no correlation, up to date, has been discovered between these entities [31]. Comparison of the previous cases and ours are described in Table 1. The average age was 52 years (range 21–79), and the vast majority were female (80%). According to Surveillance, Epidemiology, and End Results (SEER) database, PTCs usually occur during the fourth and fifth decades of life and have a 2.5 : 1 female-to-male ratio [32], while the reported mean age for PC presentation was 44 to 45 years with an even distribution between genders [33–35]. As a result, the coexistence of thyroid and parathyroid carcinoma may have an impact on the gender distribution and age presentation. Notably, the youngest patient reported had Hürthle cell thyroid carcinoma and PC [21]. Hürthle cell carcinoma is associated with poorer prognosis and higher recurrence rate in local lymph nodes [36, 37].

Almost all patients had functional PC (including our patient) as outlined in Table 2. Nonfunctional PC is much more infrequent than functional PC, and it has a decreased survival due to its absence of symptoms and perhaps to its aggressive biology [16, 39]. To our knowledge, only one case of nonfunctional PC occurring with PTM was reported [40]. Interestingly, all cases with reported PC localization including the one here reported had a preponderance for the left lower side.

A common but not diagnostic features of PC include trabecular architecture of the parenchymal cells, a thick capsule, fibrous trabeculae traversing the gland, nuclear atypia, and high mitotic rates [41–43]. Criteria for definitive diagnosis of PC consist of capsular and vascular invasion, adjacent tissues invasion, involvement of regional lymph nodes, and distant metastatic lesions. Because cell seeding can occur due to the rupture of capsule in the operative field, locally recurrent disease is not diagnostic. Our patient had a clear capsular and vascular invasion (Figures 2(b) and 2(c)), making the diagnosis of PC unequivocal. It has been reported that extracapsular vascular invasion correlates best with the diagnosis [33].

Risk factors for PC include history of neck irradiation, long-standing secondary hyperparathyroidism (HPT), end-stage renal disease, and hereditary HPT-jaw tumor syndrome. Additionally, mutation of parafibromin (HRPT2, also known as CDC73) and cyclin D1 (CCND1) plays an important role in the molecular pathogenesis of PC [1, 44, 45]. Our patient has no history of neck irradiation and has not underwent genetic testing.

Here, we describe a case of a PTH-secreting PC that had an appearance of parathyroid adenoma in imaging studies. It was suspected due to marked hypercalcemia and a very high iPTH and renal and bone disease. PTC was only diagnosed postoperatively with histological examination.

Radical surgery provides the best possibility of cure. However, more than 50% of the patients have persistent or recurrent disease. The main cause of morbidity and mortality is due to hypercalcemia complications [34] and less frequently to metastasis [46].

## 4. Conclusion

The presented case emphasizes the requirement of high clinical suspicion of parathyroid carcinoma in the presence of very high level of iPTH, marked hypercalcemia, and overt skeletal and renal involvement. The existence of any thyroid nodules should be approached and coexistence of other carcinomas should be considered. Although imaging studies assist in the localization, reliable distinction of parathyroid carcinoma from adenoma cannot be made. Radical surgery is the only effective and curative treatment and should consist of initial en bloc resection of the tumor with ipsilateral thyroidectomy and central neck dissection.

## Figures and Tables

**Table 1:** Laboratory at admission and at two months postoperative.

Laboratory values (normal range)	At admission	At two months postoperatively
Calcemia (8.6–10.3 mg/dL)	14.3	9.5
Ionized calcemia (4.36–5.2 mg/dL)	7.06	4.83
Magnesium (1.9–2.7 mg/dL)	2.1	1.94
Creatinine (0.3–0.7 mg/dL)	2	1.77
Albumin (3.5–5.7 g/dL)	3.68	3.24
Alkaline phosphatase (34–104 U/L)	381	399
PTH (12–88 pg/mL)	1160	7.4
Phosphatemia (2.5–5 mg/dL)	2.73	3.28
25(OH) vitamin D (30–100 ng/mL)	10.1	43
FT4 (0.63–1.34 ng/dL)	0.72	0.97
T3T (0.64–1.81 ng/mL)	1.07	0.79
TSH (0.3–5 mIU/L)	1.8	4.14

**Table 2:** Clinical and biochemical features of 15 patients with coexistence of parathyroid and thyroid carcinoma.

Reference	Age	Gender	Calcium (mg/dL)	PTH (pg/mL)	Parathyroid gland size (cm)	Carcinoma location	Thyroid carcinoma	Associated parathyroid disease	Surgical treatment	Outcome
Kurita et al. [38]	68	F	12.2	6300	4.2 × 3.2 × 2.4	Left lower	Papillary	None	En bloc resection	Postoperative normocalcemia
Christmas et al. [26]	62	F	Hypercalcemia	Unknown	Unknown	Unknown	Follicular	None	Unknown	Died from metastatic parathyroid carcinoma
Savli et al. [22]	47	F	Normal	Normal	Normal	Unknown	Papillary	Hyperplasia	Total thyroidectomy parathyroidectomy (excision of 2 hyperplastic glands)	Normocalcemia (1 year)
Bednarek-Tupikowska et al. [27]	42	F	15.4	1655	5 cm in diameter	Left lower	Follicular	None	En bloc resection	Persistent hypercalcemia
Schoretsanitis, 2002 [24]	55	F	14.2	>1000	3 × 3	Left lower	Papillary	None	En bloc resection	Normocalcemia (6 years)
Kern et al. [28]	54	F	Unknown	465	2.5 × 1.8 × 1.6	Right lower	Papillary and follicular	None	Right parathyroidectomy; total thyroidectomy with local lymph node resection; corticectomy in the right superior frontal gyrus	Died from intracranial metastatic parathyroid carcinoma
Lin et al. [25]	38	M	16.5	351	4 × 3 × 3	Left lower	Papillary	Two enlarged parathyroid glands on contralateral side	Total thyroidectomy and left parathyroidectomy	Normocalcemia (6 years)
Goldfarb et al. [16]	58	M	14.4	2023	3.4 × 3.3 × 2.2	Left lower	Papillary	Contralateral parathyroid adenoma	En bloc resection	Persistent hypercalcemia after resection of parathyroid carcinoma; normocalcemia after excision of contralateral parathyroid adenoma (1 year)
Marcy et al. [17]	42	F	14.1	383	1.3	Right lower	Papillary	None	Total thyroidectomy, right parathyroidectomy, and central and lateral neck dissection	Normocalcemia (14 months)
Chaychi et al. [18]	79	F	10.4	89	1.1 × 1.2 × 4.8	Left superior	Papillary	None	Total thyroidectomy and left parathyroidectomy	Normocalcemia (6 months)
Amoodi et al. [19]	48	F	Unknown	186	>5	Left lower	Papillary	None	En bloc resection	Persistent hypercalcemia after resection of parathyroid carcinoma; hypoparathyroidism after completion of parathyroidectomy
Zakerkish et al. [21]	21	M	13	1311	Unknown	Unknown	Hürthle	None	Total thyroidectomy and parathyroidectomy	Persistent hypercalcemia and died due to its complications
Song et al. [30]	45	F	17	1455	4.28 × 3.09 × 2.54	Left lower	Papillary	None	Left parathyroidectomy and left thyroid lobectomy plus left neck dissection	Persistent hypercalcemia after left parathyroidectomy; normocalcemia after left thyroid lobectomy plus left neck dissection (6 months)
Baek et al. [23]	68	F	12.8	1247	4.2 × 3.3 × 3.1	Left lower	Papillary	None	Left inferior parathyroidectomy and left thyroid lobectomy	Postoperative normocalcemia
Kuzu et al. [29]	52	F	11.4	208	Unknown	Unknown	Papillary	None	Right parathyroidectomy and right thyroid lobectomy	Postoperative normocalcemia
Our case	50	F	13.9	1160	2.4 × 1.8 × 1.4	Left superior	Papillary	None	Left inferior parathyroidectomy and left thyroid lobectomy	Postoperative normocalcemia
